# Systemic barriers to rare disease management in conflict zones: insights from a refugee with sturge-weber syndrome in Sudan

**DOI:** 10.1186/s41043-025-00845-y

**Published:** 2025-04-02

**Authors:** Awab H. Saad, Ismat B. Babiker, Sulieman H. Saad

**Affiliations:** 1 University of Medical Sciences and Technology, Khartoum, Sudan; 2 National University, Khartoum, Sudan

**Keywords:** Challenges, Rare diseases, Sturge-Weber syndrome, Multidisciplinary care, Conflict

## Abstract

Sturge-Weber Syndrome (SWS), is a rare neuro-oculo-cutaneous disorder that presents unique diagnostic and management challenges, particularly in resource-limited settings. This editorial reflects on a recent case of an undiagnosed SWS in an Ethiopian refugee patient in Sudan, highlighting systemic barriers to healthcare access during a time of war and the importance of clinical vigilance. We advocate for local and global initiatives to further enhance diagnostic capabilities, develop integrated care systems in recognition and management of such a rare and complex condition.

## Introduction

Sturge-Weber Syndrome (SWS) is a rare, non-inherited genetic condition linked to guanine nucleotide-binding protein, alpha q (GNAQ) gene mutations, causing vascular malformations in the skin, brain, eyes, and oral mucosa. It occurs in 1 in 20,000 to 50,000 live births [1]. Diagnosing and treating SWS can be particularly challenging in low-resource areas lacking advanced imaging and multidisciplinary care due to several reasons including the rarity of the condition, unfamiliarity of clinicians of such rare diseases in clinical practice, and the need for a robust multidisciplinary team to manage such complicated cases optimally.

This editorial discusses a case of a 39-year-old Ethiopian refugee in Sudan, who developed neurological features unrelated to her initial cause of hospital admission [2] and was eventually diagnosed with SWS. The purpose of this manuscript is to highlight the challenges of diagnosing and managing rare diseases such as SWS in resource-limited environments amidst the ongoing armed conflict in Sudan. This conflict has severely affected the healthcare system in Sudan as it rendered about 80% of hospitals in the country out of service and more than 65% of people lacking access to healthcare according to the World Health Organization (WHO) apart from the issues of forcible displacement, famine and overall decline in livelihood [3].

### Case overview: a challenging diagnosis in a crisis setting

This 39-year-old female patient was admitted with heart failure caused by severe anemia. However, during admission, she developed seizures, hemiplegia, aphasia, and emotional lability. A vague neurologic presentation that is not apparently related to her cause of admission. Limited access to advanced diagnostic imaging and an uncertain and incomplete patient history delayed the diagnosis of SWS. Information from her family overseas revealed two episodes of uninvestigated and untreated seizures 20 years earlier but was otherwise medically free and has no significant medical history. Repeat physical examination identified a faint port-wine stain on her left forehead—a critical diagnostic clue [4]. The patient was pregnant in her third trimester, before she suffered an abortion after the onset of her seizures during admission highlighting the burden of neurological and systemic diseases on pregnancy [5]. A brain non-contrast CT scan showed cortical calcifications in the posterior parietal region. The patient’s lesion, confirmed to be present since birth and unchanged, was diagnosed as a (PWS), ruling out a salmon patch. Considering other differential diagnoses, the patient did not exhibit any features of megalencephaly capillary malformation syndrome or Klippel-Trenaunay-Weber syndrome. Thus, based on the CT scan findings and clinical features, the diagnosis was concluded as SWS. Moreover, the patient’s late recurrence of seizures highlights the unpredictable progression of SWS and the diagnostic challenges in resource-limited settings. The management of this rare and complex case was complicated, however, available resources were fully used to mitigate her condition including, arranging possible interdisciplinary consultations, using the help of a language interpreter, delivering good counseling to the patient’s spouse and arranging follow-up visits with different specialists after discharge as well as giving recommendations for the patients referring refugee organization regarding the need for further medical assessment including ophthalmology consultation and physical rehabilitation, and speech therapy and long-term care.

### Systemic barriers to diagnosis and treatment

Managing rare diseases such as Sturge-Weber Syndrome (SWS) in conflict zones can be challenging due to limited healthcare infrastructure, lack of specialists, and insufficient diagnostic tools. In this case, the diagnosis was delayed by an uncertain history regarding early the patient’s previous seizures, unfamiliarity of clinicians with SWS itself and the rarity of the condition, language barriers, and the unavailability of timely neuroimaging. Studies have shown that many low-resource settings face critical shortages of advanced imaging equipment such as CT or MRI, exacerbating delays in diagnosing and treating medical conditions [6]. Additionally, healthcare workers often lack training and experience in identifying and managing rare conditions, which contributes to further diagnostic challenges as suggested by literature [7]. Moreover, a study that investigated the cost of managing rare diseases have found that out-of-pocket treatment costs are higher for people with rare diseases than others [8], which suggests that financial constraints can also be detrimental to the health of patients with rare conditions. Managing a complex condition such as SWS requires a multidisciplinary team and working in a scope of national and global networks specialized in rare diseases; but such services are often unavailable in limited-resource environments such as conflict zones, leading to suboptimal outcomes and jeopardy to health and outcome [9, 10], in addition, the difficulty of finding specialized and family physicians for adequate follow-up add to the challenges faced by refugees such as our patient [11].

### Addressing healthcare inequities in resource-limited and conflict zones

This case emphasizes the healthcare challenges in conflict zones, where limited access to advanced diagnostic tools such as MRI and CT angiography, along with a shortage of specialists, delays diagnosis and complicates the management of conditions such as (SWS). These gaps worsen health disparities, especially for rare diseases, which are often underdiagnosed, leading to poor outcomes for patients.

To address these issues, local and global efforts should focus on implementing advanced diagnostic tools, telemedicine services, and enhancing healthcare worker training in rare disease management. Telemedicine has been effective in bridging the specialist gap, improving diagnosis and management remotely [11]. Additionally, training local healthcare workers to identify and treat rare diseases can improve early diagnosis and care [7]. Integrated care models, where specialists collaborate for comprehensive care, are essential in such environments, and should be supported by NGOs, governments, patient advocacy groups and international organizations through infrastructure development, telemedicine and mobile health units and patient advocacy groups [12].

Thus, the need for multidisciplinary care in managing complex cases such as SWS is clear. However, in conflict zones, the lack of access to these specialists results in treatment gaps. Therefore, integrated care models, reinforced by telemedicine and other support systems, are crucial for improving outcomes for rare disease patients in such challenging settings.

### Call to action: improving healthcare systems in crisis settings

This refugee case with SWS highlights the critical need for systemic and policy-based healthcare reforms in crisis and resource-limited settings in order to manage rare diseases adequately. It also emphasizes the importance of prioritizing stronger diagnostic systems, promoting multidisciplinary care, and improving rare disease recognition in low-resource settings. A coordinated effort by governments and global health organizations is essential to build infrastructure, train clinicians, and address the unique needs of patients with rare chronic conditions [13]. Furthermore, several international projects and NGOs, such as the Rare Diseases International organization, work to prioritize rare diseases in health policies, through securing funding for sustainable resources, and raising public awareness. They also focus on improving access to treatment and healthcare, especially in resource-limited environments, by supporting initiatives for better diagnosis and community engagement [7]. Such efforts require several interventions including raising awareness, development of telemedicine platforms, deployment of mobile health units, training local healthcare providers on rare diseases, and integration of rare disease protocols into refugee health programs.

A comprehensive policy-based solution for rare diseases in refugee settings is mandatory to face such challenges specially in a limited-resource setting and it should include improving the access of refugees to healthcare, training healthcare workers on rare disease recognition, incorporating rare disease management into refugee health programs, and strengthening diagnostic infrastructure. Additionally, collaboration with governmental, local NGOs and global health organizations is vital to support these initiatives with telemedicine and expert consultation services and mobile-health units. This approach ensures more comprehensive care for refugees and patients with rare diseases and limits the faced healthcare disparities.

### Healthcare training on rare diseases: strategies and approaches

Training healthcare professionals on rare diseases involves a comprehensive approach that includes integrating rare disease education into medical curricula, specialized modules and courses about such conditions, and creating interdisciplinary pathways, along with continuing medical education programs to keep healthcare providers updated on the latest research and treatment strategies, and implementing online platforms, real case study reviews, and patient-centered education [ncl^14^]. This helps professionals understand the clinical, emotional, and social aspects of rare diseases [15]. Additionally, Collaborations with rare disease networks and expert-led training ensure accurate knowledge, while incorporating rare disease guidelines into healthcare protocols and patients’ health records may help in facilitating diagnosis and treatment. Moreover, Simulation-based training and feedback may further improve healthcare professionals’ competency, leading to better patient outcomes.

### Challenges in strengthening healthcare in crisis settings

Implementing healthcare interventions in crisis settings, especially for rare diseases, faces several challenges, including limited funding, logistical issues with telemedicine and mobile health units, and lack of infrastructure and specialized professionals in conflict areas and refugee camps. To address these obstacles, nation and worldwide collaboration efforts, prioritizing partnerships for funding, using affordable technologies, and involving the community in healthcare delivery may help. Additionally, setting policies, training healthcare workers and community leaders through continuous education and awareness can overcome cultural barriers, staff deficiencies, and treatment delay and may provide better use of resources.

### Examples of successful programs in management of rare diseases

Several successful programs have been implemented to address the challenges of managing rare diseases including the following organizations; Orphanet, a European platform that provides information and facilitates collaboration among healthcare professionals and researchers. Global Genes offers international aid and support through providing resources, training, and patient networks. National Organization for Rare Disease (NORD) in the U.S. supports patients with rare diseases with education, research funding, and access to care, while European Organization for Rare Diseases (EURORDIS) connects patient organizations and healthcare providers across Europe to improve healthcare access and research.

## Conclusion and recommendations

This editorial discusses the challenges of managing rare diseases such as Sturge-Weber Syndrome in conflict zones and resource-limited areas, highlighted by the case of an Ethiopian refugee in Sudan. It shows how barriers to diagnosis and lack of specialized care worsen outcomes for patients. The case calls for better clinical awareness, improved access to care, and more investment in healthcare infrastructure, especially in crisis settings. It also emphasizes the need for further research on the epidemiology, prevalence, and long-term effects of rare diseases in such environments. Additionally, further studies should explore the practicality and effectiveness of the suggested solutions in improving care for affected patients.

### Limitations

This editorial could be limited by the fact that it’s based on a single case study, which may not fully represent the broader challenges faced by individuals with similar or rare conditions in conflict zones, limiting the generalizability of the findings. Additionally, the editorial is focused on a single condition in a single limited-resource setting, thus, it lacks comprehensive data restricting its ability to draw more widely applicable conclusions or propose generalizable evidence-based solutions.

## Data Availability

No datasets were generated or analysed during the current study.

## References

[1] Sturge-Weber syndrome. National Organization for Rare Disorders. Available at: https://rarediseases.org/rare-diseases/sturge-weber-syndrome/. Accessed November 25, 2024.

[2] Saad AH, Omar SM, Elgilli AA, Omer IAA, Jalaleldeen MH. An atypical seizure onset and re-emergence in a refugee with an undiagnosed Sturge-Weber syndrome: a case report from a limited setting. Int Med Case Rep J. 2024;17:615–20. 10.2147/IMCRJ.S472356. 38933806 10.2147/IMCRJ.S472356 PMC11203771

[3] https://www.emro.who.int/sdn/crisis/index.html (Accessed on 12th March 2025).

[4] Ch’ng S, Tan S. Facial port-wine stains e clinical stratification and risks of neuro-ocular involvement. J Plast Reconstr Aesthet Surg. 2008;61(8):889–93. 10.1016/j.bjps.2007.05.011. 17604243 10.1016/j.bjps.2007.05.011

[5] Renukesh S, Rai L. Neurological disorders complicating Pregnancy - Focus on obstetric outcome. J Clin Diagn Res. 2016;10(12):QC06–9. 10.7860/JCDR/2016/19839.8955. Epub 2016 Dec 1. PMID: 28208940; PMCID: PMC5296513. 28208940 10.7860/JCDR/2016/19839.8955 PMC5296513

[6] Medical imaging and nuclear medicine: a Lancet Oncology Commission Hricak. Hedvig The Lancet Oncology, Volume 22, Issue 4, e136 - e172. 10.1016/S1470-2045(20)30751-8 PMC8444235 33676609

[7] Shafie AA, Chaiyakunapruk N, Supian A, Lim J, Zafra M, Hassali MAA. State of rare disease management in Southeast Asia. Orphanet J Rare Dis. 2016;11:107. 10.1186/s13023-016-0460-9. 27484654 10.1186/s13023-016-0460-9 PMC4969672

[8] Lewin Group for the Evelyn Foundation The National Economic Burden of Rare Disease Study. 2021. [(accessed on 11 March 2025)]. Available online:https://everylifefoundation.org/wpcontent/uploads/2021/02/The_National_Economic_Burden_of_Rare_Disease_Study_Summary_Report_February_2021.pdf

[9] Rare Diseases International Agreement with the WHO [Internet]. 2019. [(accessed on 12 March 2025)]. Available online: https://www.rarediseasesinternational.org/working-with-the-who/

[10] Durmus S, Yucesan E, Aktug S, Utz B, Caglayan AO, Gencpinar P, Günay C, Oktay Y, Yildirim RN, Yigit A, Ozbek U. Management of rare and undiagnosed diseases: insights from researchers and healthcare professionals in Türkiye. Front Public Health. 2025;12:1501942. 10.3389/fpubh.2024.1501942. 39911789 10.3389/fpubh.2024.1501942 PMC11795313

[11] Choudhury MC, Chaube P. Integrating rare disease management in public health programs in India: exploring the potential of National health mission. Orphanet J Rare Dis. 2022;17:43. 10.1186/s13023-022-02194-z. 35144652 10.1186/s13023-022-02194-z PMC8832777

[12] Patterson AM et al. Apr. Emerging roles and opportunities for rare disease patient advocacy groups. Therapeutic advances in rare disease vol. 4 26330040231164425. 24 2023, 10.1177/26330040231164425 10.1177/26330040231164425 PMC10184204 37197559

[13] Verma IC, El-Beshlawy A, Tylki-Szymańska A, Martins A, Duan YL, Collin-Histed T, et al. Transformative effect of a humanitarian program for individuals affected by rare diseases: Building support systems and creating local expertise. Orphanet J Rare Dis. 2022;17(1):87. 10.1186/s13023-022-02192-1. PMID: 35369888; PMCID: PMC8977120. 35369888 10.1186/s13023-022-02192-1 PMC8977120

[14] Adachi T, El-Hattab AW, Jain R, Nogales Crespo KA, Quirland Lazo CI, Scarpa M, Summar M, Wattanasirichaigoon D. Enhancing equitable access to rare disease diagnosis and treatment around the world: A review of evidence, policies, and challenges. Int J Environ Res Public Health. 2023;20:4732. 10.3390/ijerph20064732. 36981643 10.3390/ijerph20064732 PMC10049067

[15] Global Genes. (2021). Rare disease education programs. Retrieved from https://globalgenes.org (Accessed on 16th March 2025).

[16] Division Research Development and Support. Faculty of Health Sciences, Stellenbosch University, South Africa. Consent form for case reports. Version1; 2008.

